# Clinical results and surgery tactics of spinal osteotomy for ankylosing spondylitis kyphosis: experience of 428 patients

**DOI:** 10.1186/s13018-019-1371-y

**Published:** 2019-10-22

**Authors:** Zhijun Xin, Guoquan Zheng, Peng Huang, Xuesong Zhang, Yan Wang

**Affiliations:** 1 Department of Orthopaedics, General Hospital of Chinese People’s Liberation Army (301 Hospital), 28 Fuxing Rd, Beijing, 100853 China; 2 Department of Orthopaedic Surgery, Affiliated Hospital of Zunyi Medical University, Zunyi, 563000 China

**Keywords:** Ankylosing spondylitis, Spinal kyphotic deformity, Vertebral column decancellation, Pedicle subtraction osteotomy

## Abstract

**Objective:**

To report the clinical results and surgical tactics of spinal osteotomy for ankylosing spondylitis (AS) kyphosis based on the experiences of 428 patients.

**Methods:**

From January 2003 to January 2015, a total of 428 patients suffering from AS kyphosis who underwent a one- or two-level pedicle subtraction osteotomy (PSO) or vertebral column decancellation (VCD) osteotomy in our hospital were reviewed. Pre- and postoperative radiological parameters and the chin-brow vertical angle (CBVA) were measured. Intraoperative, postoperative, and general complications were recorded.

**Results:**

All patients could walk with horizontal vision and lie on their backs postoperatively. The pre- and postoperative average global kyphosis (GK) angles were corrected from 82.6 to 12.7° (*p* = 0.000) in the two-level group and from 55.8 to 9.6° (*p* = 0.000) in the one-level group, respectively. The mean sagittal vertical axis (SVA) improved from 29.4 to 8 cm (*p* = 0.000) in the two-level group and from 18.0 to 4.3 cm (*p* = 0.000) in the one-level group. The CBVA improved from 68.3 to 8.2° (*p* = 0.000) in the two-level group and from 46.2 to 4.2° (*p* = 0.000) in the one-level group. Although no major acute complications such as death or complete paralysis occurred, the complication rate was 6.5% in the one-level group and 23.6% in the two-level group.

**Conclusion:**

Spinal osteotomy, such as PSO and VCD, can improve the quality of life of AS patients as well as correct kyphotic deformities. The one-level spinal osteotomy showed a lower complication rate, while two-level spinal osteotomy was a relatively aggressive procedure that was more suitable in correcting severe AS kyphotic deformities.

## Highlights

First, the clinical results and surgical tactics of spinal osteotomy for ankylosing spondylitis (AS) kyphosis were detailed.

Second, efficacy and safety of one-level osteotomy and two-level osteotomy are systematically compared.

Third, a novel spinal osteotomy technique, vertebral column decancellation (VCD), was introduced for patients with AS with severe kyphotic deformities.

Fourth, key points of the VCD osteotomy technique were detailed.

## Introduction

Ankylosing spondylitis (AS) is a chronic spondyloarthropathy that primarily involves the spine and sacroiliac joints [1–3]. At advanced stages of AS, many cases result in spinal deformities, such as the loss of lumbar lordosis or an increase in thoracic kyphosis, which can lead to structural and functional impairments and a decrease in the quality of life [3]. Additionally, AS may be associated with severe sagittal imbalance, trunk collapse, and flexion-contracture deformities of the spine in the later stages, which may cause back pain, horizontal vision loss, or neurological deficits [4, 5]. Complications, such as walking difficulties, abdominal viscera compression, or lung dysfunction, may occur in patients with AS severe kyphotic deformities [6–8].

Surgical correction of kyphosis is necessary for many patients with AS deformities to restore sagittal balance and the ability to see straight ahead [1, 9]. However, the most effective and safe surgical procedure for AS-related symptomatic kyphotic deformities is still controversial [2, 8], and the planning processes, which have been explored to determine the ideal site and to calculate the exact angle required for an osteotomy, carry some limitations as well [10].

The purposes of this study were to report the radiographic and clinical results of spinal osteotomy for AS kyphosis patients in our single spine center and to evaluate and compare the efficacy and feasibility of a one-level osteotomy and a two-level osteotomy for correcting kyphosis secondary to AS. In addition, the surgical tactics, including the corrective angle, surgical site, and number of osteotomies, are also described.

## Materials and methods

### Patient selection

After the Institutional Review Board approved the study protocol, we retrospectively reviewed 448 AS patients who underwent spinal osteotomies, including the pedicle subtraction osteotomy (PSO) and vertebral column decancellation (VCD), for AS-related kyphotic deformities at our hospital between January 2003 and January 2015. All patients underwent evaluation by laboratory tests, including erythrocyte sedimentation rate (ESR) and C-reactive protein (CRP) tests, which were measured twice, and the patients should have the normal values before surgery. The inclusion criteria for the study were as follows: (1) 18 to 60 years of age and the preoperative diagnosis of AS was made according to the modified New York criteria [11]; (2) kyphosis in the lumbar, thoracolumbar, or thoracic spine and unable to lie flat in bed and/or look straight forward owning to the kyphotic deformities; and (3) followed up a minimum of 2 years after surgery and all of the radiographic and clinical data were complete. The exclusion criteria were as follows: (1) a history of a spinal surgery and (2) a severe concomitant disease, such as a spinal tumor or infection.

**Fig. 1:**
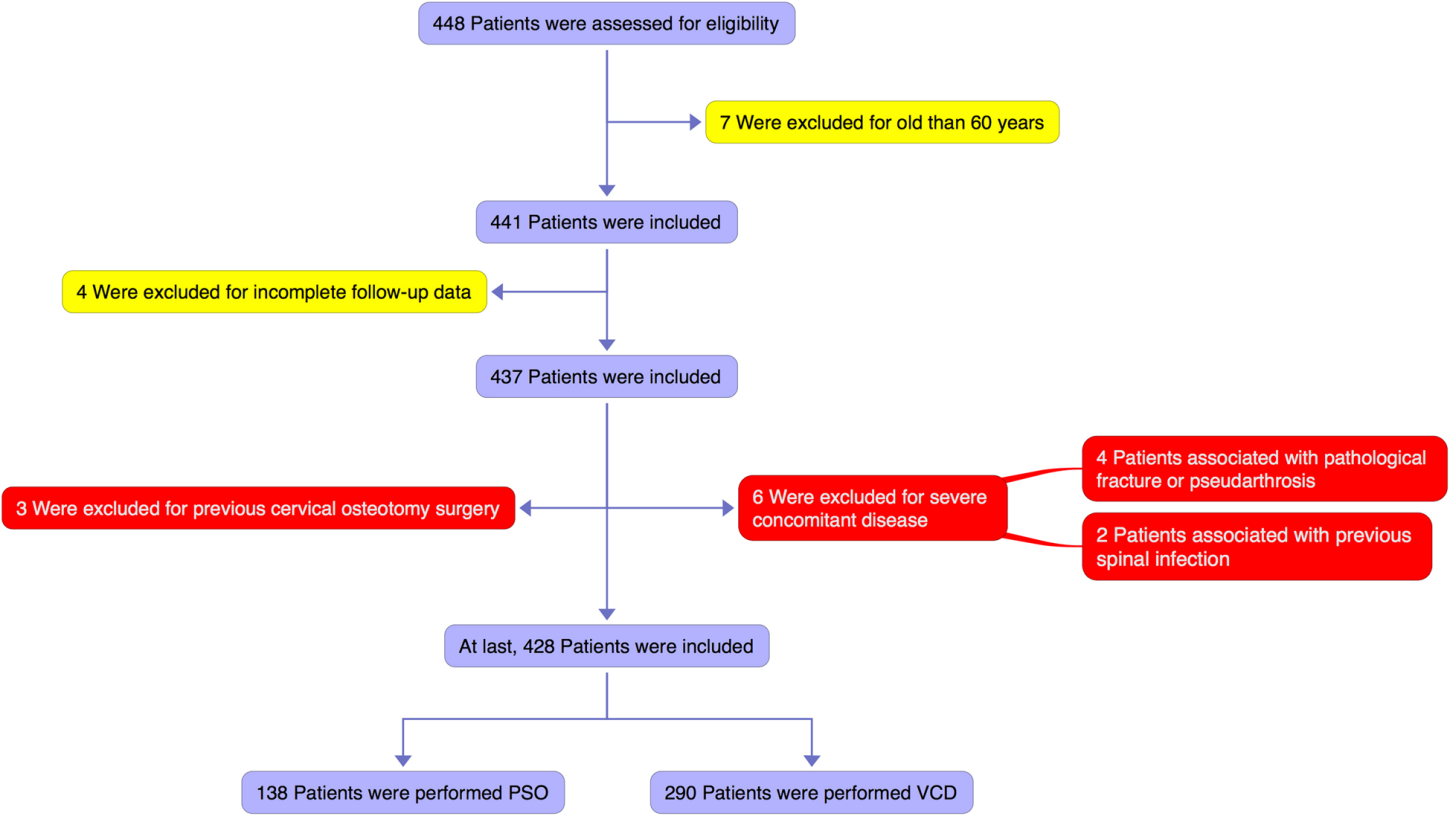
The flow diagram to demonstrate the screening process

As a result, a total of 428 patients (392 males and 36 females) with an average age of 34.8 years (range, 18–60 years) and a mean duration of AS of 14.2 years (range, 3.3–36.2 years) met the aforementioned inclusion criteria and were enrolled in this study (Fig. 1). Table 1 summarizes the clinical characteristics and radiological findings in the study.

**Table 1:** Clinical characteristics, radiological findings, and osteotomy methods in 428 AS patients

Variable	Value*
Sex	
Male	392
Female	36
Age (years)	
Mean	34.8
Range	18–60
Osteotomy method	
One-level	339
Two-level	89
Duration of AS (years)	
Mean	14.2
Range	3.3–36.2
GK (°)	
Mean	59.6
Range	32.4–110.6
SVA (cm)	
Mean	21.3
Range	6.3–49.5
CBVA (°)	
Mean	51.5
Range	28–108.1
Follow-up (years)	
Mean	2.8
Range	2.4–14

*GK* global kyphosis, *SVA* sagittal vertical axis, *CBVA* chin-brow vertical angle

*Values represent the number of patients, unless otherwise indicated

### Surgical strategies making

To choose the appropriate osteotomy approach, surgical site, and corrective angle, the following principles were taken into consideration:

1. The osteotomy site was located in the lower thoracic and upper lumbar vertebrae, usually between T12 and L3;
2. For a one-level osteotomy, the corrective angle should be no more than 40° for PSO [9, 12] or 50° for VCD [4, 13, 14]. If the required angle was larger than these angles, a two-level osteotomy was considered;
3. Two-level spinal osteotomies should not be performed with continuous segments, especially in spinal cord regions;
4. For thoracic kyphosis, we prefer the osteotomy site to be T12 or L1; for thoracolumbar or lumbar kyphosis, we prefer the osteotomy site to be L2 or L3;
5. Then, the osteotomy angle at the site was calculated based on the new osteotomy angle calculation principle [10, 15], stating that the center of gravity (CG) of the trunk should nearly be over the hip axis (HA) when the pelvis and lower extremity joints are in the neutral position.

### Surgical technique

**Fig. 2:**
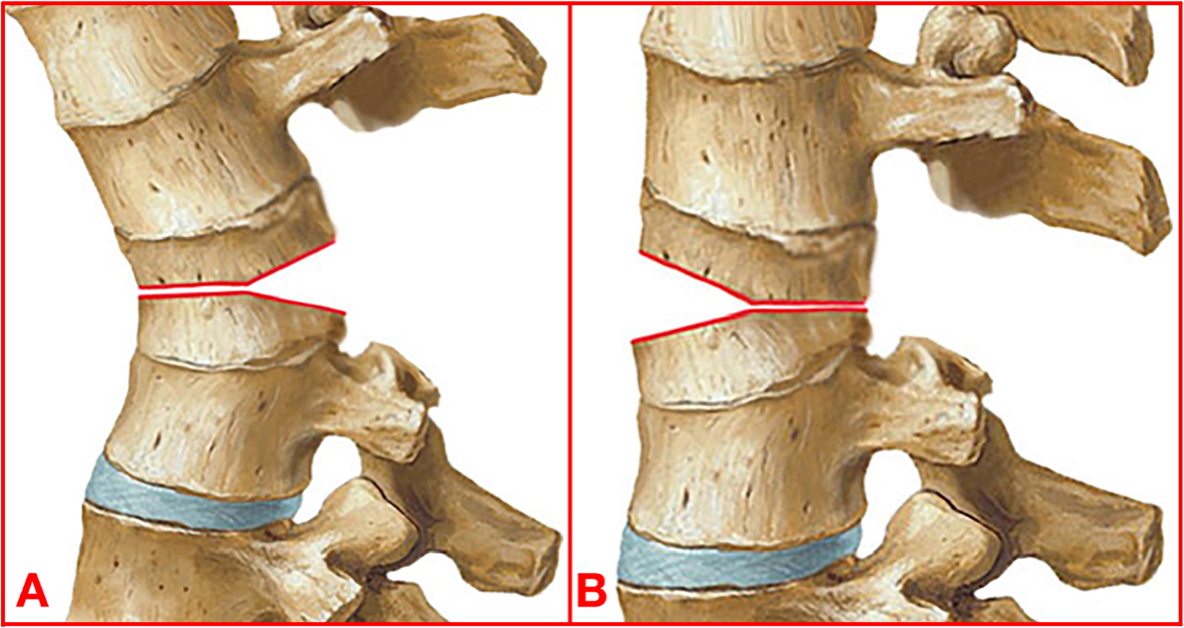
Schematic illustration of VCD osteotomy. **a** The anterior and middle column of the vertebra was removed as less as possible while the posterior column was removed completely. The osteotomy gap is “Y” shape rather than “V” shape. **b** Pressing the posterior column, the anterior column served as the hinge at the beginning and then the hinge moved to the middle column at the end

**Fig. 3:**
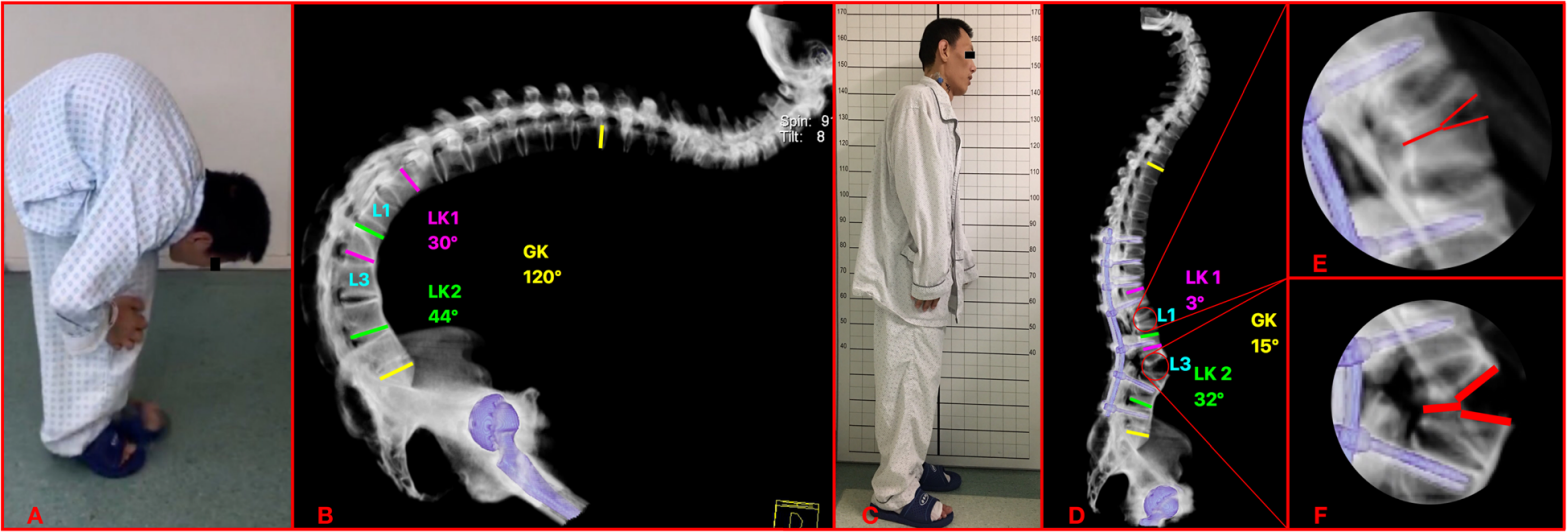
Two-level VCD osteotomy in a 38-year-old man: preoperative clinical photo (**a**) and radiographs (**b**) show 120° global kyphosis. Postoperative clinical photo (**c**) and lateral radiograph (**d**) after two-level VCD performed at L1 and L3 show his excellent sagittal alignment with the global kyphosis corrected to 15° and the osteotomy vertebrae were posterior column closing with anterior column opening just like shape “Y” (**e**, **f**). The local kyphosis of superior osteotomy vertebra (LK1) improved from preoperative 30° kyphosis (**b**) to postoperative 3° lordosis (**d**) and local kyphosis of inferior osteotomy vertebra (LK2) from preoperative 44° kyphosis (**b**) to postoperative 32° lordosis (**d**)

PSO, based on the method of Thomasen and Bridwell [16, 17], was performed in 138 patients. VCD (Figs. 2 and 3), based on the method that has been reported by our research group [13, 18], was performed in the remaining 290 patients and began with the probing and dilation of both sides of the pedicles of the osteotomized vertebrae by the pedicle probe or drill. Afterwards, a special spacer was used to enlarge the pedicle holes. Through the bilateral pedicle holes, the cancellous bone of the middle column of the osteotomized vertebra was partly removed. The anterior cortex and lateral walls of the osteotomized vertebra were thinned by a curette or high-speed drilling via both pedicle holes. Then, a laminectomy was performed at the osteotomy site. Then, the bent rods were installed into the pedicle screws above and below the osteotomy site. The posterior wall of the vertebral body and the both sides of the pedicle residual medial wall were removed using forceps or a Kerrison rongeur. A “Y”-shaped osteotomy rather than a “V”-shaped osteotomy was made by this point in the surgery. Subsequently, the middle column was closed by the gradual extension of the operating table and continuous pressure on the pedicle screws above and below the osteotomy. The hinge was on the anterior column at beginning of this procedure, and then the hinge was moved to the middle column at the end of this procedure.

Several technical advantages of VCD as a corrective technique have been described in previous studies [13, 18]. To achieve a good result, many technical tactics should be highlighted: (1) the cancellous bone of the anterior column was removed with a small curette rather than forceps or a Kerrison rongeur to reduce the risk of thoracic and ventral aorta injuries; (2) the cortex of the anterior and lateral walls was thinned and fractured linearly with a curette, ultrasonic osteotome, or high-speed drill via both pedicle holes to facilitate opening of the anterior column and closing of the middle column during the reduction; and (3) to avoid neurological damage, it was safer to perform a wide and creeping expansion laminectomy, and to avoid excessive shortening of the spinal cord.

### Postoperative management

Postoperative management was the same in all patients, and they were allowed to sit up in bed 24 h after the surgery. The drain was removed when the output decreased to < 50 ml/24 h, which was usually 3–5 days after surgery. The patients were allowed to ambulate with a custom-made plastic thoracolumbosacral orthosis (TLSO) after 3 days. If there was cerebrospinal fluid (CSF) leakage, the drainage time and time in bed should be prolonged. The TLSO was used during the first 3 to 6 months after surgery.

### Radiologic and clinical parameters

The standard radiographic measurements included global kyphosis (GK) [19], which was measured from the superior end plate of the T5 thoracic vertebra to the superior end plate of the S1 vertebra. Local kyphosis (LK) [20] was defined as the Cobb angle between the superior endplate of one vertebra above the osteotomy and the inferior endplate of one vertebra below the osteotomy (LK1: superior osteotomy vertebra; LK2: inferior osteotomy vertebra). The sagittal vertical axis (SVA) [21] was the distance between the C7 plumb line and the posterior superior corner of S1. The clinical records were reviewed for operative time, blood loss, complications, and chin-brow vertical angle (CBVA) [22], which was defined as the angle measured between a line from the brow to the chin and a line from the chin to the vertical axis while the patient stood with his or her hips and knees extended.

### Statistical analysis

Statistical analyses were performed using SPSS (version 18.0; SPSS, Inc., Chicago, IL, USA). Comparisons of the pre- and postoperative spinal sagittal parameters and CBVA were performed with a paired sample *t* test. The patients were divided into two groups according to the number of osteotomy segments, and the operative time, the blood loss volume, and the rate of complications were compared. Values of *p* < 0.05 were considered statistically significant.

## Results

All patients could walk with horizontal vision and lie on their backs postoperatively. A one-level osteotomy was performed in 339 patients, and a two-level osteotomy was performed in the remaining 89 patients (Table 1). No mortalities or any major neurologic complications occurred during the follow-up period; however, 32 patients suffered one or two complications, including CSF leaks (*n* = 21, 9 in the two-level group and 12 in the one-level group), transient neurological deficits (*n* = 3, in the two-level group), vascular laceration bleeding (*n* = 1, in the two-level group), infections (*n* = 2, 1 in the two-level group and 1 in the one-level group), postoperative low back pain (*n* = 5, 2 in the two-level group and 3 in the one-level group), spinal rod broken (*n* = 3, 2 in the two-level group and 1 in the one-level group), distally pedicle screws pull out (*n* = 4, 2 in the two-level group and 2 in the one-level group), and non-fusion at the osteotomy site (*n* = 4, 3 patients associated with Andersson’s lesion preoperatively), as shown in Table 2, and there were significant differences between two groups in complications of CSF leaks and neurologic deficit (*p* < 0.05).

**Table 2:** Frequency of complications in two groups

Complication	Frequency		*χ* ^2^	*p*
	One-level group (*n* = 339)	Two-level group (*n* = 89)		
CSF leaks	12	9	5.19	0.023
Neurologic deficit	0	3	–	0.009^a^
Vascular laceration bleeding	0	1	–	0.208^a^
Surgical site infection	1	1	–	0.373^a^
Low back pain	3	2	0.26	0.610
Rod broken	1	2	–	0.111^a^
Pedicle screws loosening	2	2		0.192^a^
Pseudarthrosis	3	1		0.608^a^

^a^Fisher’s exact test

The preoperative, postoperative, and final follow-up radiological and clinical outcomes of the 428 patients are shown in Table 3. All patients demonstrated changes in the pre- and postoperative radiological parameters and the CBVA, while no significant differences were demonstrated in these parameters between the postoperative and final follow-up. The pre- and postoperative average GK angles were 82.6° and 12.7° (*p* < 0.05), respectively, in the two-level group and 55.8° and 9.6° (*p* < 0.05), respectively, in the one-level group. The CBVA improved from 68.3 to 8.2° (*p* < 0.05) in the two-level group and from 46.2 to 4.2° (*p* < 0.05) in the one-level group. The mean SVA improved from 29.4 to 8 cm (*p* < 0.05) in the two-level group and from 18.0 to 4.3 cm (*p* < 0.05) in the one-level group. All of these parameter changes demonstrated significant differences between the one- and two-level osteotomy groups (*p* < 0.001). The operative time, blood loss, and complication rate data are shown in Table 3. The average operative time was 253 min for the one-level group and 331 min for the two-level group, the average operation-related blood loss was 537 ml in the one-level group and 1132 ml in the two-level group, and the complication rate was 6.5% in the one-level group (22 of 339 patients) and 23.6% in the two-level group (21 of 89 patients). The results demonstrated that the operative time, blood loss, and frequency of complications were significantly higher in patients who underwent the two-level osteotomy than in patients who underwent the one-level osteotomy (*p* < 0.001; Table 3).

**Table 3:** Pre-, intra-, and postoperative clinical and radiologic parameters of the patients

Variable	One-level osteotomy (*n* = 339)	Two-level osteotomy (*n* = 89)	*p*
Operation time (min)	253 ± 51.2	331 ± 85.3	< 0.001*
Blood loss (ml)	537 ± 121.3	1132 ± 417.2	< 0.001*
Complication rate (%)	6.5%	23.6%	< 0.001*
GK (°)			
Pre-op	55.8 ± 21.3	82.6 ± 29.2	
Post-op	9.6 ± 6.2^#^	12.7 ± 12.1^#^	
Final follow-up	11.2 ± 7.8^#^	13.5 ± 11.8^#^	
GK correction	44.6 ± 13.5	69.1 ± 17.4	< 0.001*
SVA (cm)			
Pre-op	18.0 ± 8.9	29.4 ± 8.5	
Post-op	4.3 ± 5.1^#^	8 ± 4.6^#^	
Final follow-up	5.2 ± 5.0^#^	9.7 ± 5.5^#^	
SVA correction	12.8 ± 4.7	19.7 ± 3.7	< 0.001*
CBVA (°)			
Pre-op	46.2 ± 10.9	68.3 ± 21.5	
Post-op	4.2 ± 3.3^#^	8.2 ± 7.9^#^	
Final follow-up	4.7 ± 3.1^#^	9.3 ± 8.4^#^	
CBVA correction	41.5 ± 7.8	57.0 ± 13.1	< 0.001*

*GK* global kyphosis, *LK* local kyphosis, *SVA* sagittal vertical axis, *CBVA* chin-brow vertical angle

*Compared with one-level osteotomy group, *p* < 0.05 and statistically significant

^#^Compared with preoperation, *p* < 0.05 and statistically significant

## Discussion

Surgical correction, such as a spinal osteotomy, may be the appropriate option for the treatment of AS patients with a severe kyphosis deformity [8, 9, 23]. As proven by our research group, spinal osteotomies not only corrected the sagittal deformity but also significantly increased pulmonary function [24, 25], digestive function by relieving abdominal viscera compression [19, 26], cardiac function [27], and sexual activity [28] of patients with AS.

Adequate preoperative planning of the surgery is critical for sagittal plane corrective osteotomies of the spine in AS patients. Suk [22] and Van Royen [29] used the CBVA and biomechanical and mathematical methods for planning the correction angle and the osteotomy site, but these methods carry some limitations [10, 30]. Thus, we have explored a new osteotomy angle calculation method according to an analysis of the CG of the trunk [10, 15]. Based on our extensive clinical experience, we found that the hilus pulmonis (HP) is located over the HA in normal subjects, so we chose the HP as the reference point for the center of CG of the trunk. Thus, if we shifted the HP over the hip axis, the exact angle required for a spinal osteotomy in patients with AS kyphosis can be calculated [10].

Theoretically, an osteotomy at the apex vertebra should achieve a better corrective effect [27, 31, 32]. With the same correction angle, an osteotomy at the lower level vertebra can achieve greater safety [33]. Therefore, we prefer to perform osteotomy at the lumbar or thoracolumbar spinal regions. Usually, we do not perform an osteotomy at L4 or L5 because L4 and L5 are not the apex vertebrae of lumbar lordosis [10], and fusion to the sacrum with a short lever arm on the distal part of fusion will result in discomfort or an inability to sit on the floor [32]. In view of these abovementioned considerations, we suggest that most osteotomy sites are located at the second and third lumbar vertebrae because the third vertebra is the apex of the lumbar spine and the second vertebra is usually near the thoracolumbar kyphosis. In addition, the second and third vertebrae are usually located below the conus medullaris, which means an osteotomy can be performed more safely. For thoracic hyperkyphosis, the osteotomy site can also be T12 or L1 because T12 and L1 are near the apex vertebrae.

In our opinion, a two-level spinal osteotomy at the spinal cord region is not recommended. First, satisfactory reconstruction of lumbar lordosis is necessary because the loss of lumbar lordosis is usually co-existent with hyperkyphosis of the thoracolumbar and thoracic spine. Second, a spinal osteotomy in a non-spinal cord region is relatively safe, and an osteotomy in the lumbar region can allow a relatively large operating space and correction angle [23]. At the same time, a continuous two-level spinal osteotomy is not recommended because excessive shortening of the area may result in buckling of the dura and spinal cord, which is very dangerous [34]. Thus, for severe thoracolumbar kyphotic deformities, the ideal combination of osteotomy sites is L1 and L3. If the apex of the kyphosis is located above T12, we may choose T12 and L2 as the osteotomy sites [23].

Several spinal osteotomy techniques, including the Smith-Petersen osteotomy (SPO) [35], PSO [16], and VCD [13, 18], have been performed for the treatment of AS kyphotic deformities. However, the most effective and safe surgical procedure for an AS-related kyphosis deformity is still controversial [2, 9]. An SPO is more suitable for a flexible kyphosis and deformities without ossification of the anterior column of the spine [36, 37]. PSO is a technically demanding procedure with relatively severe surgical trauma and a high risk of complications [37]. VCD was first described in 2011 by our research group as an operative technique for the treatment of patients with a sharp angular spinal deformity [18]. Due to its many advantages as an osteotomy technique, VCD has been performed for and is an effective and excellent treatment option for kyphotic deformities in patients with AS [13, 14].

In the current study, the total complication rate was 10.1% (43 complications in 32 patients) for all 428 patients, and there were no major acute complications, such as death or complete paralysis. The patients were divided into two groups according to the number of osteotomy segments (one- and two-level osteotomy groups), and the complication rate was significantly higher in the two-level osteotomy group than in the one-level osteotomy group. Therefore, a two-level osteotomy can provide more extensive correction than a one-level osteotomy, but it has a higher complication rate.

In our experience, CSF leakage, which is the most common complication in the literature [13, 23], is difficult to avoid when the dura is extremely thin due to chronic inflammation adhering to the surroundings, especially to the ossified ligamentum flavum [1, 9]. If a dural tear occurs intraoperatively, prompt management of the leak with a gel sponge or muscle and myofascia should be made, a drainage tube should be left until the output rate falls below 50 ml/24 h, and the duration of drainage and bedrest should be prolonged if needed. There are three causes of vascular damage: the osteotomy procedure direct injury, the excessive elongation injury of the anterior column, and osteotomy stump stab. Therefore, gentle manipulation during surgery and a linear fracture at the anterior cortex of the osteotomized vertebrae are important in order to avoid vascular damage. In addition, avoiding excessive stretching of the anterior column also reduces the risk of thoracic and ventral aorta injury. To avoid neurological damage, it is safer to perform a creeping expansion laminectomy and to avoid excessive shortening of the spinal posterior column. Many investigators have reported that pseudarthrosis or rod breakage can occur when the osteotomy is performed through an area that is not previously fused at the time of osteotomy [36, 38]. Thus, we suggest that a sufficient bone graft must be achieved to facilitate better bony fusion and better stability in the corrected position during the osteotomy. For preventing infection, adhering to the principles of asepsis during and after surgery and sufficient wash of the operation area will help. There are some other complications that have been reported, such as paralytic ileus [39], which resolved after the insertion of a Levin tube and oral intake restriction, and blindness, possibly caused by local extrusion during the operation, which may be avoided if individuals such as non-operative personnel pay more attention to protecting the patient’s eyes [40]. Because of the high complication rate of spinal osteotomies, the indication should be carefully assessed, the surgery should be performed by an experienced spine surgeon who has mastered various types of deformity correction techniques, and the role of non-operative assistants is also of importance, especially when positioning patients and during reduction [23].

Based on the large number of AS-related kyphotic deformities treated by spinal osteotomies in our single spine center, we have many key recommendations to ensure the successful performance of several spinal osteotomy techniques:

1. A detailed preoperative surgical plan based on radiological and clinical evaluations is critical. A greater understanding of the neural structures and deformity types of the spine can be gained. The method, location, and range of the osteotomy can be determined, and the length and fixation point of the internal fixation can be selected.
2. Bleeding can be controlled with bipolar electrocautery, with the application of absorbable hemostatic gauze and a gelatine sponge and/or with controlled hypotension during the operation. At the same time, three methods can be used to replenish blood loss: (1) cell saver, which re-transfuses the patient’s own blood; (2) blood from the blood bank is given to the patient when necessary; and (3) for the first 6 h, blood from the suction drains is collected and transfused using a postoperative cell saver [18].
3. Accurate fixation of the pedicle screws, sufficient length fixed of the spine (at least two segments above and below the designated osteotomy site), and a high success rate of disposable nailing should be ensured to provide sufficient and reliable fixation strength.
4. Extended central laminectomy should be performed to the adjacent lamina above and below the osteotomy level to ensure that there are no impingements on the dural sac and nerve roots; then, the neurologic elements can be visualized directly during closure.
5. Optimal prebending of the spinal rod should be conducted and sufficient pressure should be applied to the spinal curve to prevent the breaking of rods and pedicle screws being pulled out.
6. For severe deformities, gentle and continuous bilateral symmetrical compression forces should be applied on the rods (one proximal and one distal to the osteotomy) at the same time during closure and supplemented by the gradual extension of the folding operating table to achieve position reduction.

## Conclusions

In this study, we reported the results of two types of spinal osteotomies for a large number of patients with AS kyphosis, focusing on the surgical strategy, technical aspects, complications, and correction outcomes. In addition, we compared the results of the one-level osteotomy with the results of the two-level osteotomy. The results demonstrate that both PSO and VCD are effective osteotomies in correcting kyphotic deformities in AS patients. In addition, the one-level spinal osteotomy showed a lower complication rate, while the two-level spinal osteotomy was a relatively aggressive procedure that was more suitable in correcting severe AS kyphotic deformities.

## Data Availability

The patients’ data were collected in the Chinese PLA General Hospital. The datasets used and/or analyzed during the current study are available from the corresponding author on reasonable request.

## Abbreviations

AS: Ankylosing spondylitis
CBVA: Chin-brow vertical angle
CG: Center of gravity
CSF: Cerebrospinal fluid
GK: Global kyphosis
HA: Hip axis
HP: Hilus pulmonis
LK: Local kyphosis
PSO: Pedicle subtraction osteotomy
SPO: Smith-Petersen osteotomy
SVA: Sagittal vertical axis
TLSO: Plastic thoracolumbosacral orthosis
VCD: Vertebral column decancellation
